# Extensive intramuscular lipoma of the thigh with Parosteal features: a case report of a rare 39-cm tumor

**DOI:** 10.1093/omcr/omag093

**Published:** 2026-06-08

**Authors:** Alhassan Hamed Alhassan, Mohammed Ganim, Mohamed Alhadi, Abdelaziz Elshafie, Basil Jalal Aldain Zain Alabdin, Fatima Almahadi Elbala Suliman, Mona I A Elagib, Sami Awad Elkarim Mohammed Elfadul, Monadhil Awad

**Affiliations:** Department of Diagnostic Radiology, College of Medical Radiologic Sciences, Karary University, Khartoum, Sudan; Research Department, National Center for Gastrointestinal and Liver Diseases, Khartoum, Sudan; Department of Radiology and Medical Imaging, Collage of Medicine, Alzaiem Alazhri University, Khartoum, Sudan; Department of Radiology and Medical Imaging, Collage of Medicine, Omdurman Islamic University, Khartoum, Sudan; Department of Surgery, Collage of Medicine, The National Ribat University, Khartoum, Sudan; Department of Histopathology, National Center for Gastroenterology and Liver Diseases, Kassala, Sudan; Department of Histopathology, National Center for Gastroenterology and Liver Diseases, Kassala, Sudan; Department of Radiology and Medical Imaging, University of Kordofan Faculty of Medicine and Health Sciences, Sudan; Department of Radiology and Medical Imaging, University of Khartoum, Faculty of Medicine, Khartoum, Sudan

**Keywords:** Giant lipoma, intramuscular, thigh, surgical excision, histopathology

## Introduction

Lipomas are the most frequent benign mesenchymal tumors arising from adipose tissue [1]. They usually present as slow-growing, soft, and mobile subcutaneous masses with no associated pain, most commonly affecting adults between the ages of 40 and 60 [2]. While most lipomas remain small and localized, a subset can grow to exceptional sizes—termed giant lipomas—which are defined either as lesions exceeding 10 cm in diameter or weighing more than 1000 grams [3]. These large tumors, though histologically benign, may cause functional and cosmetic deformities, compress adjacent structures, or raise clinical suspicion of malignancy, particularly well-differentiated liposarcomas [4].

Giant lipomas have been described in various anatomical regions, including the neck, shoulder, thigh, and back, with flank or lateral trunk presentations being relatively uncommon. Surgical excision remains the treatment of choice, both for definitive diagnosis and symptom relief. Complete excision with capsule removal is essential to minimize recurrence risk, which remains low for benign lipomas. Recurrence is uncommon but has been reported in up to 19% of incompletely excised intramuscular lipomas. Long-term follow-up is therefore advisable [5] However, surgical challenges increase with tumor size, anatomical depth, and proximity to vital structures, warranting careful preoperative planning.

We report a case of a 65-year-old male with a giant intramuscular lipoma of the right thigh, measuring approximately 39 × 10 × 13.5 cm, which caused functional impairment and required complete surgical excision.

## Case presentation

A 65-year-old male presented with a progressively enlarging swelling in the right thigh. The patient initially experienced mild discomfort with no gait disturbance 6 years ago but did not seek medical attention or use any medications. Over the following years, the swelling gradually increased in size, eventually causing difficulty or pain during walking, which prompted hospital evaluation. His medical history was unremarkable, with no diabetes, hypertension, prior surgeries, or familial similar conditions. He was a non-smoker and did not consume alcohol.

On physical examination, a large, non-tender soft tissue mass occupied the right thigh. The overlying skin appeared normal, without erythema, warmth, or other changes. No regional lymphadenopathy was detected.

Ultrasound examination revealed a large intramuscular lesion extending along the anterior, medial, and lateral aspects of the thigh, measuring 30 × 10 × 18 cm. The mass abutted the femoral shaft, was predominantly hyperechoic with some heterogeneous areas, and maintained smooth borders. No internal vascularity was noted. CT scan confirmed a well-defined, homogeneous, hypodense, non-enhancing soft tissue mass located between the right femur and surrounding musculature, measuring 39 × 10 × 13.5 cm. The lesion had a density of approximately −100 Hounsfield Units, which consistent with adipose tissue, and thin internal septations without nodular enhancement and with thin non-enhancing septations and a few foci of calcification, consistent with a fatty mass (Fig. 2).

**Figure 1:**
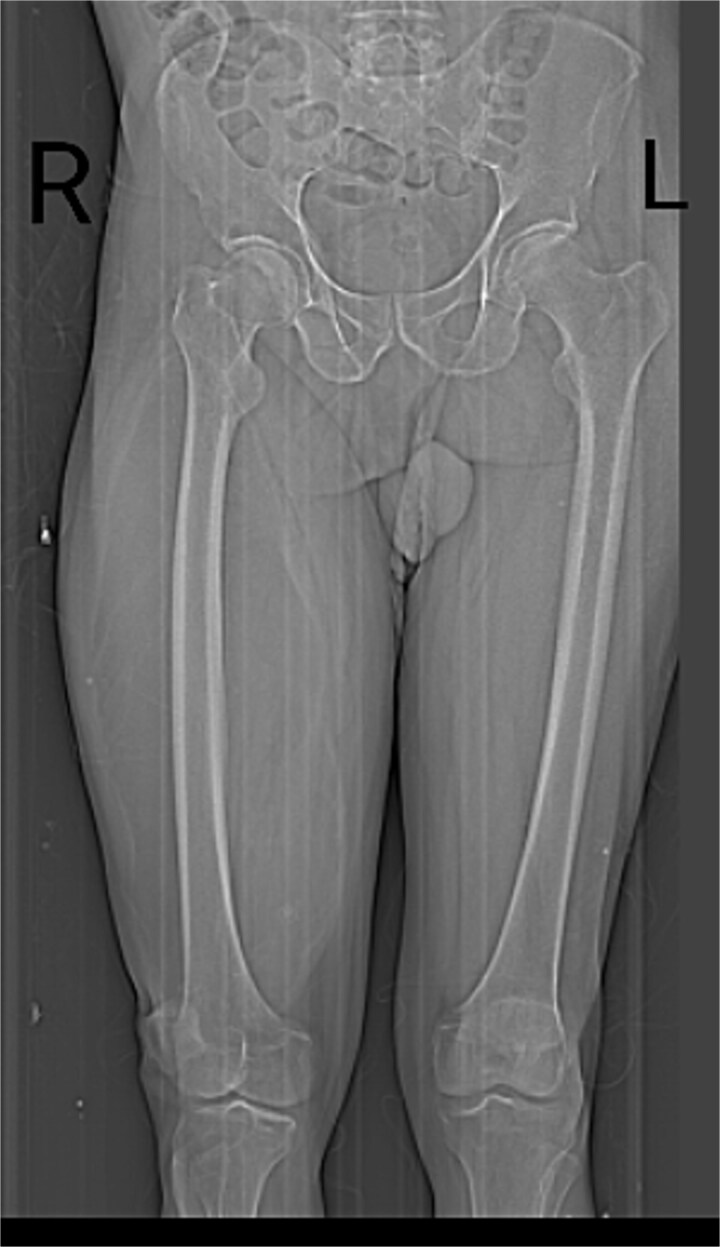
Large smooth soft tissue mass with most of the right thigh with intact femur, no periosteal reaction or distraction changes.

**Figure 2:**
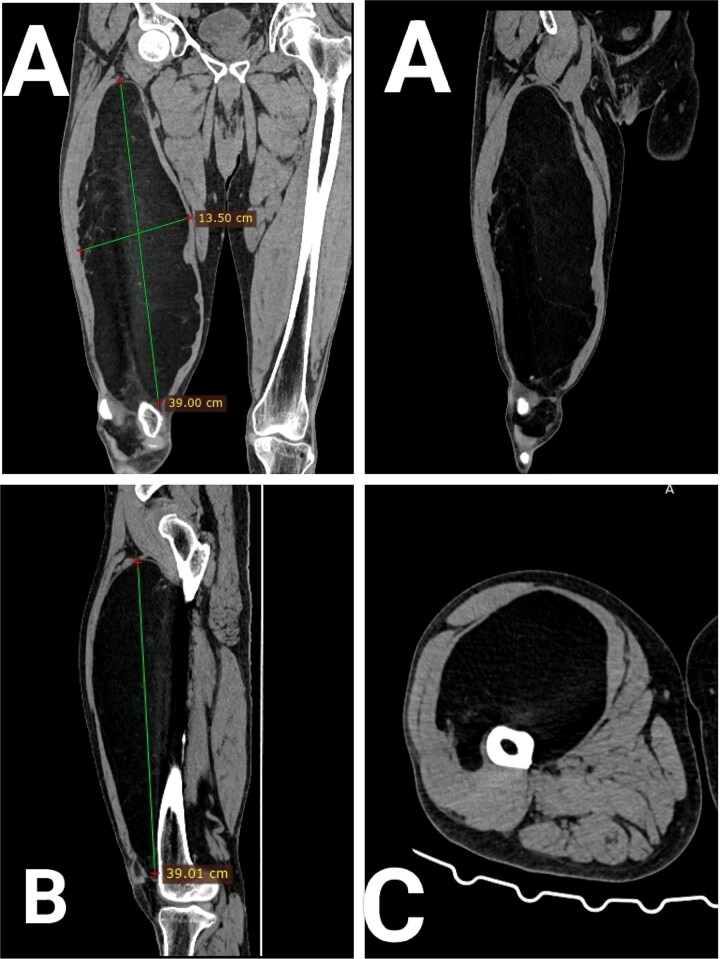
(A) Coronal, (B) Sagittal, (C) Axial sections for non-contrast CT scan demonstrating lipoma (HU below −100) within & expanding right Vastus intermedius muscle pushing adjacent Vastus lateralis, Vastus medialis & rectus femoris muscles, with no medial and anterior surface of femur reflecting parosteal component.

**Figure 3:**
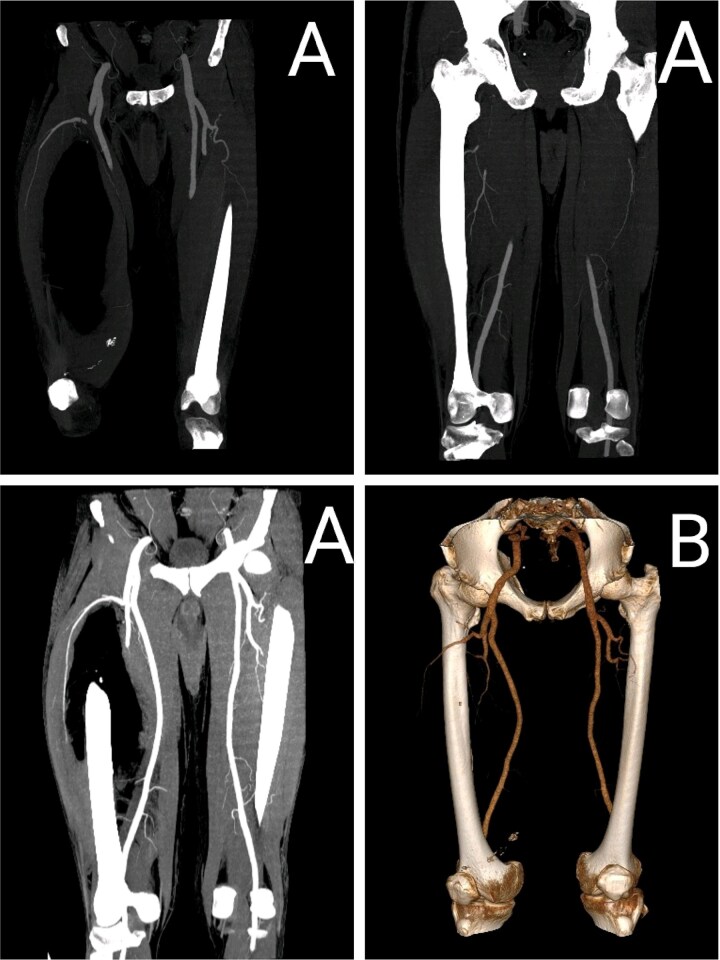
(A) Coronal images, (B) 3D volume rendering image showing Giant lipoma within right Vastus intermedius muscle without encasement or compression of superficial & deep femoral arteries. The read arrows demonstrate few foci of calcifications, no intact bone, no periosteal reaction or bone distraction.

**Figure 4:**
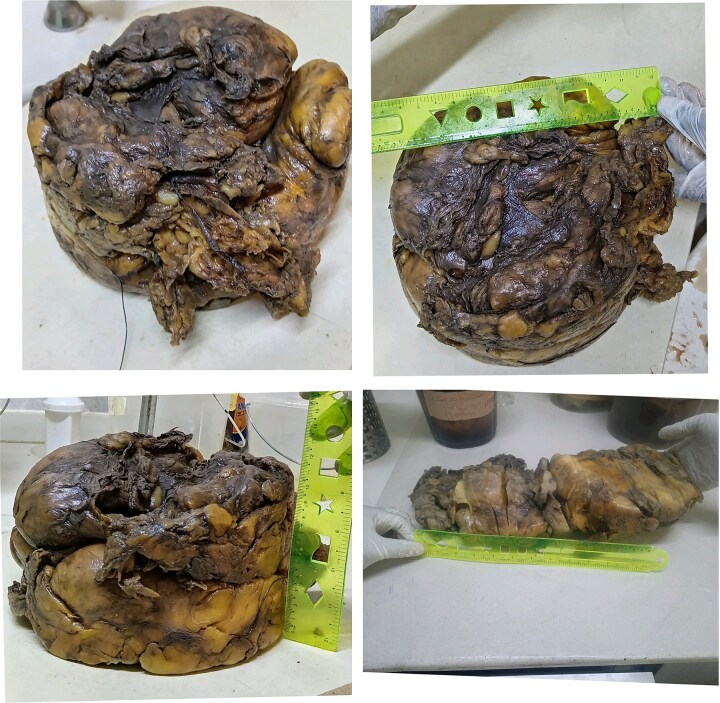
Tumour after surgical excision, received 2 masses of fatty tissue, measuring collectively 30 × 20 × 10cm cut sections yellowish.

**Figure 5:**
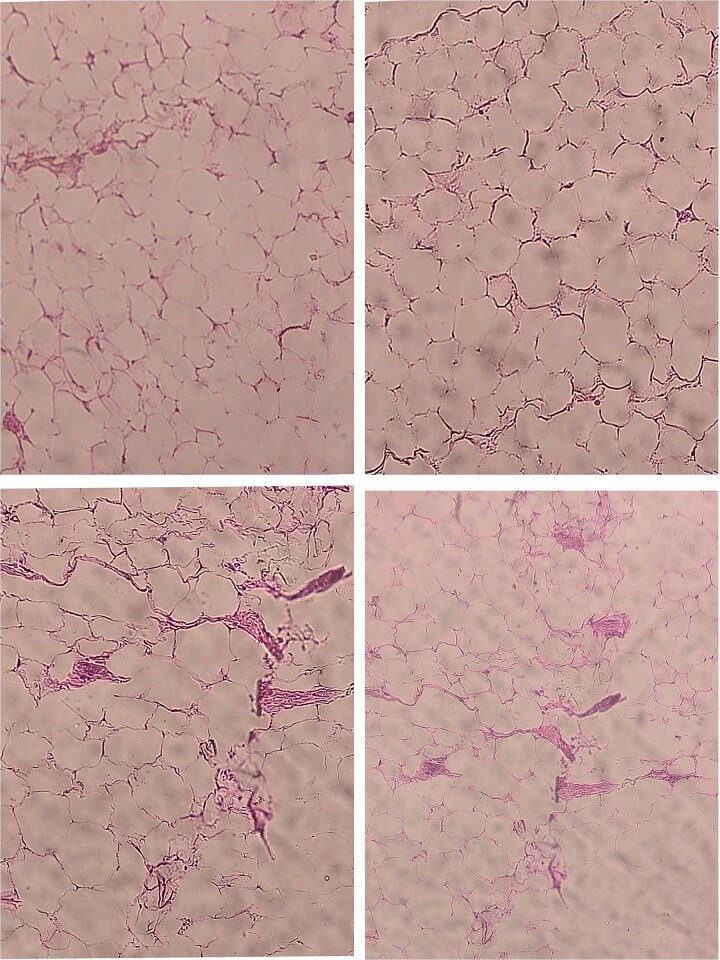
Section shows lobules of bland-appearing mature adipose tissue separated by vascularized thin fibrous septae (H&E, ×10).

Surgical excision was planned under spinal anaesthesia with the patient in a supine position. A longitudinal incision was made over the right thigh, and the subcutaneous tissue and muscle layers were carefully dissected to expose the intramuscular lipoma. Due to the large size of the mass and its intramuscular location, excision was performed in two stages to allow safe surgical access, ensure careful dissection around surrounding muscle and neurovascular structures, and minimize the risk of intraoperative complications such as bleeding or tissue injury. Hemostasis was secured, the wound was closed in layers, skin closure was achieved with nylon sutures, and a drain was inserted. The excised mass measured approximately 39 × 10 × 13.5 cm and was sent for histopathological examination. The procedure was completed without intraoperative complications.

Postoperative recovery was uneventful. The drain was removed, and the patient showed marked improvement, being discharged in good condition. The patient was able to ambulate independently, reported no pain, and had a well-healed surgical site without signs of infection or hematoma. Histopathological examination revealed two fatty tissue masses, collectively measuring 39 × 20 × 10 cm (Fig. 4). Microscopically, lobules of mature adipose tissue separated by thin, vascularized fibrous septa were observed, with unremarkable skeletal muscle fibers, foci of calcification, and chronic inflammatory cells. No evidence of malignancy was detected, confirming the diagnosis of a giant intramuscular lipoma (Fig. 5).

## Discussion

Lipomas are common benign mesenchymal tumors, typically subcutaneous and small.—Intramuscular variants are rare and may reach exceptional sizes, mimicking malignancy. They typically arise in the subcutaneous tissue of the trunk or extremities and remain relatively small, often less than 5 cm [1]. By contrast, intramuscular lipomas are uncommon, representing only a small fraction of all cases, and they are usually more challenging to detect due to their deep location [6]. The term ‘giant lipoma’ refers to tumors exceeding 10 cm in diameter or 1 kg in weight. However, reports of intramuscular lipomas larger than 20 cm are exceedingly rare [3].

Our case is exceptional in this context, with the tumor reaching approximately 40 cm in greatest dimension, making it one of the largest intramuscular lipomas described to date.

The clinical importance of such giant lesions lies in their diagnostic overlap with malignant soft tissue tumors, particularly well-differentiated liposarcoma. Both entities may present as large, slow-growing, and painless masses, and imaging alone may not reliably distinguish them [7]. Radiologically, giant intramuscular lipomas can mimic other soft tissue lesions. Differential diagnoses include well-differentiated liposarcoma, which may present as a large, slowly growing fatty mass with septations and occasional nodular areas; intramuscular hemangioma, which often shows vascular channels and may demonstrate contrast enhancement; and fibrolipoma, which contains both fibrous and fatty components. Careful imaging evaluation combined with histopathology is essential to distinguish these entities and guide appropriate management [7].

In addition to differentiating benign from malignant fatty tumors, it is also essential to accurately classify lipomas according to their anatomical relationship with adjacent structures.

An important diagnostic consideration in this case was distinguishing between a parosteal and a periosteal lipoma. Parosteal lipomas arise adjacent to the bone surface without originating from the periosteum, whereas periosteal lipomas arise directly from the periosteum and typically show continuity with the underlying cortex [8]. In our case, imaging demonstrated a well-defined fatty mass between the right femur and surrounding musculature without cortical continuity or periosteal reaction, supporting the diagnosis of a parosteal lipoma. This distinction is important for accurate diagnosis and surgical planning.

Ultrasound remains the first-line imaging modality. In the presence of atypical features, it is essential to perform cross-sectional imaging [4].

MRI is considered the gold standard. It can detect signs suggestive of sarcomatous transformation, such as thick, enhancing septa or walls, and the presence of non-fatty, enhancing tissue components.

It also provides an accurate assessment of the relationships with adjacent structures.

Imaging plays a crucial role in the evaluation of giant intramuscular lipomas. In this case, CT scan revealed a well-defined, homogeneous, hypodense, non-enhancing soft tissue mass located between the right femur and surrounding musculature, measuring 39 × 10 × 13.5 cm. The lesion had a density of approximately −100 Hounsfield Units, consistent with adipose tissue, with thin internal septations and a few foci of calcification, without nodular enhancement. Hounsfield Unit values are particularly helpful in characterizing fatty lesions, supporting the diagnosis of a lipoma and aiding in differentiation from other soft tissue masses such as well-differentiated liposarcomas or hematomas. Unfortunately, MRI imaging, which could have provided additional soft tissue characterization and anatomical detail, was not available for this case.

Histopathology remains the gold standard for differentiating between lipoma and well-differentiated liposarcoma. Lipomas are composed of mature adipose tissue without cellular atypia, whereas well-differentiated liposarcomas show varying degrees of atypia, hyperchromatic nuclei, and sometimes fibrous septa. In our patient, histology revealed mature adipose tissue without atypia, confirming the diagnosis of a benign lipoma.

Surgical excision is the definitive treatment, with the primary goals being complete removal of the tumor and preservation of surrounding functional anatomy. In the case of giant intramuscular lipomas, surgery can be technically demanding due to the tumor’s proximity to important neurovascular structures and the risk of significant bleeding. Moreover, because intramuscular lipomas tend to infiltrate muscle fibers, incomplete resection carries a risk of recurrence. Careful preoperative planning, including detailed imaging and intraoperative identification of tumor boundaries, is therefore critical. In our case, the mass was successfully excised in its entirety with preservation of muscle integrity, and the patient had a favourable postoperative course.

Compared with previously published cases, most giant lipomas described in the literature range from 10 to 20 cm, with common locations including the shoulder, neck, and lower limb [9–11]. Reports of tumors approaching or exceeding 30 cm are exceedingly uncommon, and very few involve an intramuscular origin. Our case therefore contributes to the limited body of evidence on the behaviour and management of extremely large intramuscular lipomas. The magnitude of this lesion emphasizes the importance of considering benign tumors in the differential diagnosis of massive soft tissue growths, even when the size suggests malignancy.

This case underscores several important learning points: (1) Giant intramuscular lipomas, though rare, can reach extreme sizes and mimic malignancy; (2) CT imaging, including HU values, is useful in characterizing fatty lesions, though contrast-enhanced studies or MRI provide superior anatomical detail; (3) Complete surgical excision requires careful preoperative planning due to proximity to neurovascular structures; and (4) Histopathology remains essential to confirm benignity and guide follow-up, particularly to monitor for recurrence in cases of incomplete excision.

## Conclusion

This case highlights the rare occurrence of an exceptionally large intramuscular lipoma, measuring 40 cm, which was successfully treated with complete surgical excision. It underlines the diagnostic challenges in differentiating giant lipomas from liposarcomas, the technical difficulties associated with their removal, and the importance of documenting such unusual presentations to guide clinicians when faced with similarly rare tumors.

## References

[1] Silistreli OK, Durmuş EU, Ulusal BG. et al. What should be the treatment modality in giant cutaneous lipomas? Review of the literature and report of 4 cases. Br J Plast Surg 2005;58:394–8. 10.1016/j.bjps.2004.09.005 15780237

[2] Kolb L, Yarrarapu SNS, Ameer MA. et al. Lipoma. 2023 Aug 8. In: StatPearls [Internet]. StatPearls Publishing: Treasure Island (FL), 2025.

[3] Fnini S, Hassoune J, Garche A. et al. Lipome géant de la main: présentation d'un cas clinique et revue de la littérature [Giant lipoma of the hand: case report and literature review]. Chir Main 2010;29:44–7. 10.1016/j.main.2009.11.006 20116318

[4] Muhib M, Abidi SLF, Ahmed U. et al. Use of radiologic imaging to differentiate lipoma from atypical lipomatous tumor/well-differentiated liposarcoma: systematic review. SAGE Open Med 2024;12:20503121241293496. 10.1177/20503121241293496 39526094 PMC11549689

[5] Salam GA . Lipoma excision. Am Fam Physician 2002;65:901–4. 11898962

[6] McTighe S, Chernev I. Intramuscular lipoma: a review of the literature. Orthop Rev (Pavia) 2014;6:5618. 10.4081/or.2014.5618 25568733 PMC4274454

[7] Zafar R, Wheeler Y. Liposarcoma. 2023 Mar 20. In: StatPearls [Internet]. StatPearls Publishing: Treasure Island (FL), 2025.

[8] Burt AM, Huang BK. Imaging review of lipomatous musculoskeletal lesions. SICOT J 2017;3:34. 10.1051/sicotj/2017015 28474576 PMC5418895

[9] Sanchez FL, Arreola Peralta RJ, Sanchez Alvarez S. et al. Giant lipoma on the left shoulder: a case report. Cureus. 2024;16:e63067. 10.7759/cureus.63067 39055435 PMC11269270

[10] Du A, Wang H, Dai J. et al. Case report: a case report of excision of giant lipoma in the posterior neck. Front Oncol 2024;14:1395130. 10.3389/fonc.2024.1395130 38800402 PMC11116667

[11] Litchinko A, Cherbanyk F, Menth M. et al. Giant gluteal lipoma surgical management. BMJ Case Rep 2019;12:e229842. 10.1136/bcr-2019-229842 PMC6700553 31413054

